# Tissue-Based Transcriptomic Profiling of Gastrointestinal Graft Versus Host Disease Reveals Immune and MicroRNA Dysregulation

**DOI:** 10.3390/ijms27052513

**Published:** 2026-03-09

**Authors:** Sakhila Ghimire, Jean Norden, Rihab Gam, Clare Lendrem, Ernst Holler, Anne M. Dickinson, Rachel E. Crossland

**Affiliations:** 1 Department of Internal Medicine III, University Medical Centre, 93053 Regensburg, Germany; sakhila.ghimire@klinik.uni-regensburg.de (S.G.);; 2 Translational and Clinical Research Institute, Newcastle University, Newcastle upon Tyne NE2 4HH, UKanne.m.dickinson@alcyomics.com (A.M.D.)

**Keywords:** GvHD, HSCT, molecular profiling, MicroRNA, transplantation

## Abstract

Gastrointestinal acute graft-versus-host disease (GI aGvHD) remains a leading cause of non-relapse mortality after allogeneic hematopoietic stem cell transplantation (HSCT). Current diagnostic methods rely on invasive procedures with limited sensitivity. While circulating biomarkers have been proposed, little is known about the local transcriptomic landscape within inflamed GI tissue. We performed integrated profiling of mRNA and microRNA expression in colonoscopically resected GI biopsies from n = 8 HSCT patients, including n = 3 with histologically confirmed GI aGvHD and n = 5 without. Using NanoString nCounter technology, we quantified 770 immune-related mRNAs and 799 mature human microRNAs. Differential expression analysis, pathway enrichment, cell type deconvolution, and machine learning–based biomarker prioritisation were conducted to define disease-specific molecular signatures. GI aGvHD was marked by upregulation of inflammatory genes (e.g., *IL1B*, *IL17RA*, *HLA-DRA*) and immune-regulatory microRNAs (e.g., *miR-155-3p*, *miR-223-3p*), alongside downregulation of epithelial and anti-inflammatory markers (*ST6GAL1*, *THBS1*, *miR-1915-3p*, *miR-145-5p*). Enrichment analyses revealed activation of IL2/STAT5, JAK/STAT3, TCR signalling, and antigen presentation pathways. Machine learning identified *LCN2*, *CXCL13*, and *miR-1269b* as top-ranked biomarker candidates. Cell deconvolution showed increased M0 macrophage and decreased dendritic cell signatures in aGvHD tissue. This is the first study to integrate mRNA and microRNA profiling in GI tissue using NanoString technology to characterise the immune and epithelial transcriptomic landscape of aGvHD. Our findings reveal dysregulated immune pathways, altered myeloid cell populations, and novel biomarker candidates, offering tissue-specific insights into disease pathogenesis and potential diagnostic targets. Larger validation studies and functional assays are warranted to confirm clinical utility.

## 1. Introduction

Acute graft versus host disease (aGvHD) remains the most significant cause of non-relapse mortality following allogeneic hematopoietic stem cell transplantation (HSCT), with approximately 40% of patients developing grade II-IV aGvHD and associated mortality rates reaching 75% in treatment-refractory cases [1]. Among affected organs, the gastrointestinal (GI) tract is particularly vulnerable, with lower GI involvement occurring in approximately 30% of transplant recipients, and associated with severe clinical manifestations, poor prognosis, and limited treatment options [2,3].

GI aGvHD arises from a complex cascade of immune events initiated by donor T cell activation in response to host antigen-presenting cells (APCs), often triggered by mucosal barrier disruption during conditioning regimens. This mucosal damage facilitates the translocation of microbiota, their PAMPs and metabolic products, further amplifying local and systemic inflammation through cytokine release and immune cell recruitment. As such, transplant clinicians routinely encounter GI disorders and complications before, during and after HSCT, which account for a significant part of the morbidity following HSCT [4]. Clinical outcome is therefore closely related to the clinical management of these complications.

Clinically, early and accurate diagnosis of GI aGvHD is critical but remains challenging due to the non-specific nature of associated symptoms. Indeed, GI aGvHD may present as severe diarrhoea, anorexia or vomiting [5], all of which may be associated with differential diagnoses following HSCT, such as bacterial or viral infections, drug toxicity or neutropenic enterocolitis [4]. While diagnostic advances such as endoscopy and imaging have improved clinical assessment, these approaches can be invasive, costly, and limited in availability, with standard endoscopic biopsy showing suboptimal diagnostic sensitivity [6]. Recent investigations into advanced imaging techniques have highlighted the ongoing need for improved non-invasive diagnostic approaches. The economic burden of repeated invasive procedures, combined with their associated morbidity, underscores the critical need for molecular biomarkers that can inform on the onset, severity, or outcome of GI aGvHD [7]. Among the most well-validated serological biomarkers for acute GvHD are Reg3α and soluble ST2 (sST2), which form the basis of the MAGIC algorithm probability (MAP) score developed by the Mount Sinai Acute GVHD International Consortium (MAGIC) [8]. These biomarkers have demonstrated strong predictive value for non-relapse mortality and treatment response, particularly in gastrointestinal involvement. However, it is important to note that Reg3α and sST2 reflect not only GI-specific damage but also broader systemic inflammatory activity, underscoring the need for complementary tissue-based markers that more directly reflect local immune regulation within the GI tract.

Advances in transcriptomic analytical approaches, including mRNA and microRNA profiling, have revealed promising molecular signatures associated with aGvHD across multiple organ systems, diagnosis and prediction of aGvHD [9,10,11]. MicroRNAs are small non-coding RNAs that regulate gene expression post-transcriptionally and are known to modulate immune responses, inflammation, and tissue injury [12]. Emerging evidence suggests that specific microRNAs, including miR-153-3p, miR-29a, miR-146a, miR-100, miR-181a, and miR-441, may serve as both biomarkers and therapeutic targets in GvHD, with some enhancing disease risk while others appear protective [13,14]. Despite advances in serum-based biomarker studies, there remains a significant gap in our understanding of local transcriptomic alterations within affected GI tissues, particularly regarding integrative analyses of miRNA–mRNA networks in situ.

In this study, we performed comprehensive molecular profiling of colonoscopically resected gut biopsy samples collected from HSCT patients at the onset of GI symptoms. Using NanoString nCounter technology, we simultaneously assessed the expression of over 770 immune-related mRNAs and 799 mature human microRNAs in matched biopsies. We performed differential expression analysis, gene set enrichment, pathway profiling, and cell type deconvolution to identify transcriptomic signatures associated with histologically confirmed GI aGvHD. Furthermore, we applied multi-algorithm machine learning approaches to prioritise high-ranking biomarker candidates and integrated microRNA and mRNA expression data to uncover putative regulatory interactions.

Together, this integrative tissue transcriptomic approach provides new insight into the molecular pathology of GI aGvHD and identifies novel candidate biomarkers with potential clinical utility. By profiling gut tissue directly, this study addresses a critical unmet need in transplant medicine and lays the groundwork for future diagnostic and therapeutic strategies targeting tissue-specific immune dysregulation in aGvHD.

## 2. Results

### 2.1. Clinical Details of the Patient Cohorts

**Figure 1:**
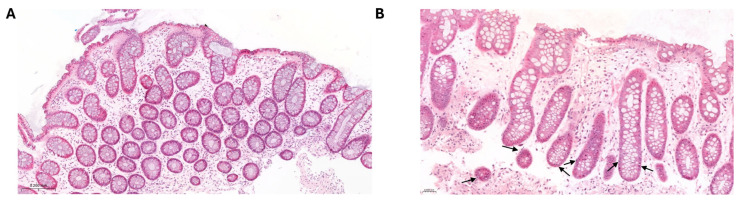
Examples of Histological Non-GvHD and GvHD Colon Biopsies (H&E Staining) (**A**) Representative cross-section of colon tissue without GvHD (Lerner grade 0), showing an intact epithelial layer and well-preserved crypt architecture with no visible apoptotic bodies. (**B**) Representative cross-section of colon tissue with GvHD, displaying multiple apoptotic bodies at the base of crypts as indicated by arrows.

Eight adult patients transplanted between 2010 and 2013 at the University Medical Centre Regensburg (Regensburg, Germany) were molecularly profiled (Table 1). There were 6/8 (75%) male and 2/8 (25%) female patients receiving 7/8 (87%) male and 1/8 (13%) female-derived transplants, with female-to-male disparity in 3/8 (38%) cases. All patients (8/8; 100%) received reduced intensity conditioning (RIC) and the majority (7/8; 87%) received a matched unrelated donor (MUD) transplant. Patients were transplanted at an average age of 50 yrs (range 24–63), and 5/8 (63%) patients or donors tested cytomegalovirus positive. There was a range of underlying diseases (Table 1). At the time of the last follow-up, 4/8 (50%) patients were deceased and 3/8 (38%) relapsed (of which 2/3 (67%) were deceased). Five (3/8; 38%) of the patients developed histological GI aGvHD grade II or above. An exemplary H&E staining for GvHD and no-GvHD GI tissue is shown in Figure 1. Non-GvHD tissue shows intact epithelium with well-preserved crypts with no visible apoptotic bodies (Figure 1A). GvHD tissue shows multiple apoptotic bodies, especially at the base of the crypts (Figure 1B). All clinical characteristics of the cohort are shown in Table 1. Given the small cohort size, all machine learning and biomarker prioritisation analyses were performed in a hypothesis-generating framework.

### 2.2. Immune Gene Expression Profiling and Phenotypic Correlations in GI aGvHD

**Figure 2:**
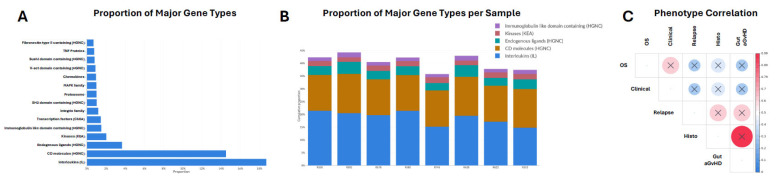
Immune gene expression composition and phenotypic correlations. (**A**) Bar plot showing the relative proportion of immune-related gene categories among expressed transcripts across all gastrointestinal biopsy samples. Gene categories were defined based on HGNC/KEGG annotations. Proportions represent the percentage contribution of each category to the total immune gene expression detected. (**B**) Stacked bar plot illustrating the distribution of major immune gene categories within each individual sample, highlighting inter-sample variability in immune gene composition across patients with differing clinical and histological phenotypes. (**C**) Correlation matrix depicting pairwise associations between clinical phenotype (clinical GI aGvHD), histological diagnosis, relapse status, and overall GI aGvHD classification. Circle size corresponds to the absolute Spearman correlation coefficient, with red indicating positive correlations and blue indicating negative correlations.

To characterise the immune transcriptional landscape of GI tissue, we examined the distribution of major immune-related gene classes and their relationships with clinical and pathological phenotypes (Figure 2). Analysis of overall gene class composition revealed that interleukins (IL) represented the largest proportion of expressed immune genes (>18%), followed by CD molecules (>14%) (Figure 2A). Additional enriched categories included endogenous ligands, kinases, and immunoglobulin-like domain-containing genes, while categories such as fibronectin-type III domains, TNF family members, and V-set domain-containing genes were comparatively less abundant (Figure 2A). This distribution indicates a transcriptional profile dominated by cytokine-mediated signalling and immune cell surface markers, consistent with the inflammatory milieu characteristic of GI aGvHD.

Assessment of immune gene class composition at the individual sample level demonstrated that interleukins and CD molecules consistently accounted for the largest proportion of expressed immune genes across both aGvHD and control GI tissues (Figure 2B). Despite this shared dominance, variation in the relative contribution of additional gene categories, including immunoglobulin-like domain–containing genes and kinases, was observed between samples, reflecting inter-sample heterogeneity in immune activation and signalling within the GI tract.

To explore associations between molecular and clinical features, correlation analysis was performed across analysed phenotypes (Figure 2C). Strong positive correlations were observed between clinical GI aGvHD and overall survival, as well as between histological GI aGvHD grade and relapse status, and between gut aGvHD classification and relapse. These relationships highlight concordance between tissue-level pathology and clinical outcomes, and support the biological relevance of the immune transcriptional patterns captured in this dataset.

Overall, this initial analysis demonstrates an immunologically active transcriptional environment in GI tissue during aGvHD, driven primarily by cytokine signalling and immune cell marker expression, and identifies clinically relevant associations between immune gene expression profiles and disease-related phenotypes.

### 2.3. Differential Gene Expression Reveals Immune Pathway Enrichment in GI aGvHD

To further dissect the molecular profiles of GI aGvHD, we performed unsupervised clustering, functional enrichment, and dimensionality reduction analyses on the most variable transcripts (microRNAs and immune genes) profiled in GI biopsies.

**Figure 3:**
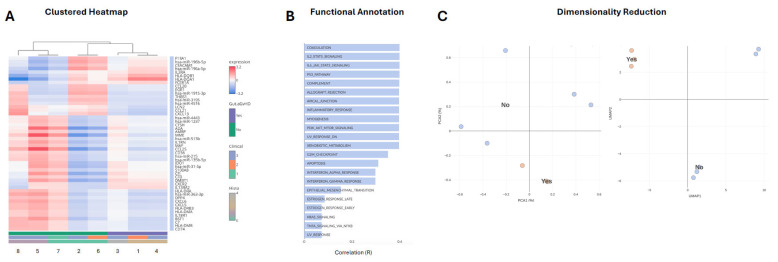
MicroRNA and Immune Gene Expression Profiling. (**A**) Heatmap of the top 50 most variable transcripts (immune genes and microRNAs) across gastrointestinal biopsy samples. Expression values were log-transformed, scaled (z-score by gene), and visualised using two-way hierarchical clustering of both genes and samples (Euclidean distance, Ward’s linkage). Red indicates relatively higher expression and blue indicates relatively lower expression compared with the cohort mean. Sample annotations indicate histological GI aGvHD status (Yes/No) and clinical grading. (**B**) Functional enrichment analysis of the gene module represented in panel A. Enriched biological pathways were identified by correlating gene expression patterns with curated gene sets from MSigDB, Gene Ontology, and KEGG databases. Bars represent correlation coefficients (R), with higher values indicating stronger association between the observed gene module and the annotated pathway. Prominent enrichment of inflammatory, immune signalling, and allograft rejection pathways is observed. (**C**) Dimensionality reduction of combined immune gene and microRNA expression profiles according to GI aGvHD status. Principal component analysis (PCA, left) and Uniform Manifold Approximation and Projection (UMAP, right) demonstrate separation of samples by histological GI aGvHD status (Yes vs. No), indicating distinct transcriptional signatures associated with disease presence.

Unsupervised hierarchical clustering revealed a distinct transcriptional pattern that segregated samples according to histological GI aGvHD status (Yes vs. No) (Figure 3A). Samples from patients with aGvHD exhibited elevated expression of genes involved in antigen presentation and immune activation (e.g., HLA-DMB, HLA-DRA, CD74), alongside inflammatory mediators (e.g., IL1B, CXCL5, CCL21, DPP4) (Figure 3A). Several immune-related microRNAs (e.g., *miR-31-5p*, *miR-135b-5p*, *miR-215*) were also differentially expressed within this cluster, suggesting integrated regulation of inflammatory pathways at transcriptional and post-transcriptional levels (Figure 3A). In contrast, samples without histological evidence of aGvHD displayed relatively lower expression across these markers.

Functional enrichment analysis of the identified gene module highlighted significant representation of inflammatory and immune signalling pathways, including IL2/STAT5, IL6/JAK/STAT3, complement activation, and allograft rejection pathways (Figure 3B). Additional enrichment of interferon responses, apoptosis, and cellular stress pathways likely reflects ongoing immune-mediated tissue injury within the gastrointestinal mucosa during active aGvHD.

Dimensionality reduction analyses further supported discrimination of samples based on GI aGvHD status (Figure 3C). Both PCA and UMAP projections demonstrated clear separation of aGvHD-positive and aGvHD-negative samples, indicating that combined immune gene and microRNA expression profiles capture biologically meaningful differences associated with disease presence. Together, these analyses suggest that GI aGvHD is characterised by a distinct and coordinated immune transcriptional signature with potential discriminatory relevance.

### 2.4. Differential Expression Analysis Distinguished GI aGvHD

To identify transcriptomic features associated with GI aGvHD, we performed differential expression analysis of both microRNAs and immune-related mRNAs in histological aGvHD (grade II) (n = 3) vs. no/mild aGvHD (0-I) (n = 5) patients.

**Figure 4:**
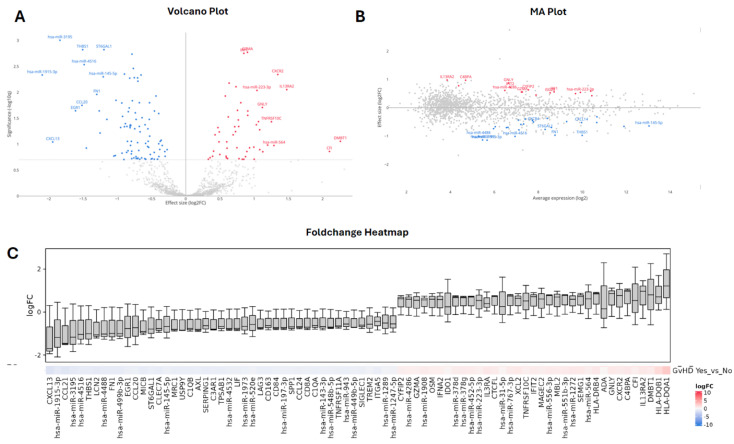
Differential Expression Analysis of MicroRNA and Immune-Related mRNAs. (**A**) Volcano plot depicting effect size (log_2_ fold change, x-axis) versus statistical significance (−log_10_ adjusted p-value, y-axis) for combined microRNA and immune-related mRNA features. Adjusted p-values were derived following multiple testing correction. Transcripts meeting the significance threshold are highlighted, with upregulated features in GI aGvHD shown in red and downregulated features shown in blue, while non-significant features are shown in grey. Selected biologically relevant transcripts are labelled to aid interpretation. (**B**) MA plot showing log_2_ fold change (y-axis) as a function of average expression (log_2_, x-axis) across all samples. This representation illustrates the relationship between transcript abundance and differential expression, highlighting that both highly and moderately expressed features contribute to the observed GI aGvHD-associated transcriptional changes. Transcripts meeting the significance threshold are highlighted, with upregulated features in GI aGvHD shown in red and downregulated features shown in blue, while non-significant features are shown in grey. Selected biologically relevant transcripts are labelled to aid interpretation. (**C**) Ranked fold change distribution of differentially expressed microRNAs and immune-related mRNAs. Boxplots represent the distribution of log_2_ fold change values across samples for each transcript, ordered from most downregulated to most upregulated in GI aGvHD. Colour shading reflects direction and magnitude of change, with blue hues indicating downregulation and red hues indicating upregulation in GI aGvHD relative to controls. This panel summarises the overall structure and variability of transcript-level changes underlying panels A and B.

**Figure 5:**
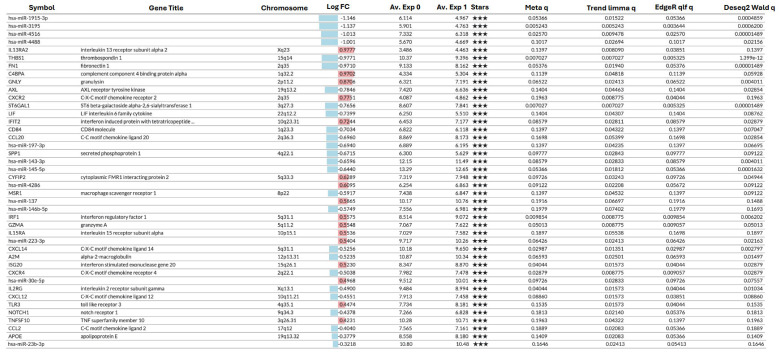
Differentially Expressed Genes and MicroRNAs. The table shows differential expression analysis using four commonly accepted methods: T-test (standard, Welch), limma (no trend, trend, voom), edgeR (QLF, LRT), and DESeq2 (Wald, LRT), and combines the statistical results using a meta.q value that represents the highest q value among the methods. Positive FC differences are indivated in red, and negative differences in blue. The number of stars indicate how many methods identified as significant.

This analysis identified 42 significantly differentially expressed transcripts between histological aGvHD and non aGvHD samples, comprising 16 upregulated and 26 downregulated features in aGvHD (Log_2_ FC Range = −1.15–0.98) (Figure 4A and Figure 5). These included 13 microRNAs (4 upregulated, 9 downregulated; Log FC Range = −1.15–0.61) and 29 immune-related mRNAs (12 upregulated, 17 downregulated; Log FC Range = −0.98–0.98) (Figure 5), indicating dysregulation of both transcriptional and post-transcriptional regulatory layers in GI aGvHD tissue.

The volcano plot (Figure 4A) illustrates the magnitude and statistical significance of these changes, highlighting a subset of transcripts with both large effect sizes and strong statistical support. Notably, immune activation-associated genes such as CEBPB, GZMB, IL17RA and IFI30 were among the most strongly upregulated features, while several transcripts involved in immune regulation and epithelial function, including ST6GAL1 and CTSE, were downregulated in aGvHD.

The MA plot (Figure 4B) demonstrates that differentially expressed transcripts spanned a broad range of expression levels, indicating that disease-associated changes were not restricted to either low- or high-abundance features. Several of the most strongly upregulated genes in aGvHD, including CEBPB, GZMB, IL17RA, were also relatively abundant, supporting their robust detectability and potential biological relevance. In parallel, inflammatory microRNAs such as miR-223-3p and miR-155-3p were upregulated at intermediate-to-high expression levels, consistent with their established roles in immune activation. Conversely, downregulated transcripts included ST6GAL1, CTSE, and microRNAs miR-4516, miR-3195, and miR-1915-3p, which were evident across a range of expression intensities.

To further visualise the overall structure of these changes, a ranked FC heatmap of all transcripts passing filtering thresholds, ranked by log2 fold change, highlighted distinct expression patterns between aGvHD and non-aGvHD samples, across both microRNA and mRNA classes (Figure 4C). Transcripts such as CXCL13, CTSE, and ST6GAL1 were consistently downregulated in aGvHD, while HLA-DRA, IFI30, and IL17RA were upregulated, collectively reflecting immune activation within GI aGvHD tissue (Figure 4C).

Together, these data provide evidence for coordinated dysregulation of immune-related genes and regulatory microRNAs in GI aGvHD, and identify candidate molecular features that may serve as biomarkers or mechanistic contributors of disease pathology.

### 2.5. Identification of Potential Biomarkers for GI aGvHD

**Figure 6:**
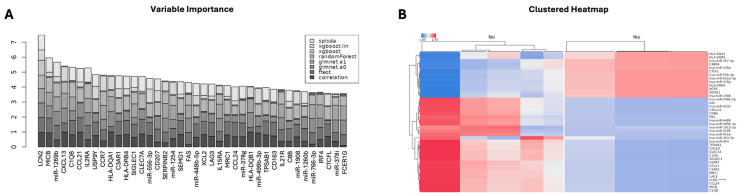
Biomarker Identification. (**A**) Variable importance plot ranking top transcripts by cumulative importance across seven feature selection methods: sPLS-DA, linear and tree-based XGBoost, random forest, glmnet (α=0 and α=1), F-test, and correlation. Higher bars indicate features consistently ranked as highly discriminatory across methods. Key biomarkers include LCN2, MICB, CXCL13, IL2RA, and HLA-DQA1. (**B**) Heatmap indicating the expression pattern of the genes and microRNAs across the phenotypic histological aGvHD groups (yes vs. no), for 40 of the most likely biomarkers. The most strongly identified biomarker is indicated by *****. Unsupervised heatmap clustering was performed using the ward.D2 method with Euclidean distance. Expression is scaled across rows (blue = low, red = high).

To understand which genes and microRNAs were influencing the histological aGvHD phenotype (Yes vs. No), we calculated a variable importance score for each feature using a multi-method machine learning algorithm strategy. The top 50 scores were calculated for cumulative ranking by the algorithms to identify possible biomarkers (Figure 6A). The top-ranking biomarkers included LCN2, MICB, miR-1269b, CXCL13, CLQB, and CCL21, which consistently scored highly across multiple machine learning frameworks. These genes and microRNAs represent examples of known immune regulators. Importantly, several transcripts, such as CXCL13, IL2RA, and HLA-DQA1, also appeared as differentially expressed in differential expression analyses (Figure 6A,B) (Figure 5), strengthening their potential utility as robust candidate biomarkers.

Unsupervised hierarchical clustering demonstrated clear segregation of samples based on aGvHD status, driven by distinct expression profiles of a core set of transcripts, including HLA-DQA1, CTGF, miR-767-3p, miR-378d, and CEBPB, which were consistently upregulated in aGvHD (Figure 6B). Conversely, samples from non-GvHD patients showed higher expression of immune-modulatory or epithelial maintenance transcripts such as miR-4488, miR-3195, miR-4516, F11, and CLEC7A (Figure 6B).

This combined machine learning and clustering approach not only validated potential candidate biomarkers identified through differential expression, but also highlighted multi-analyte signatures potentially capable of discriminating GI aGvHD status.

### 2.6. Gene Expression and Pathway Profiling Distinguishes GI aGvHD

To further contextualise the transcriptional changes observed in GI aGvHD, we performed gene set enrichment analysis (GSEA) and pathway over-representation analyses using annotations from WikiPathways, Reactome and Gene Ontology (GO). These complementary approaches were used to identify coordinated biological processes underlying the differentially expressed immune genes identified in Figure 4.

**Figure 7:**
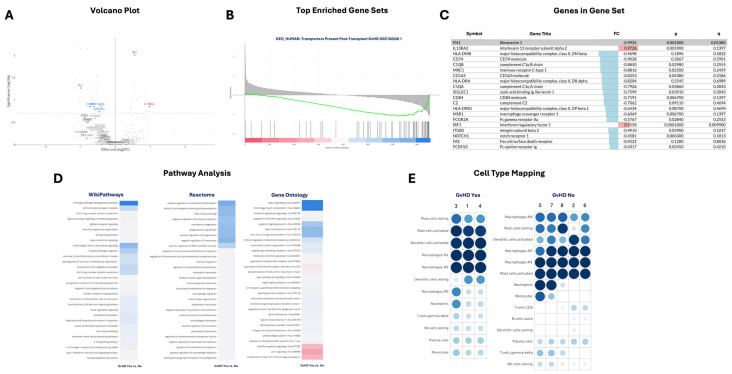
Pathway, Gene Set and Cell-Type Enrichment Analysis. (**A**) Volcano-plot showing differentially expressed immune-related genes comparing aGvHD vs. non-aGvHD GI biopsies. The x-axis shows effect size (log_2_ fold change) and the y-axis shows statistical significance (−log_10_ adjusted p-value). Significantly upregulated genes in aGvHD are shown in red and downregulated genes in blue; selected representative genes are labelled to highlight key immune pathways. (**B**) Gene Set Enrichment Analysis (GSEA) illustrating enrichment of the “Activation of the Pre-synaptic Phase of TCR Signaling” gene set in aGvHD samples. Genes are ranked by differential expression, with black vertical bars indicating the position of gene set members within the ranked list. The green curve represents the running enrichment score (ES). A leftward shift of the peak indicates enrichment in aGvHD samples. (**C**) Table summarising the leading-edge subset of genes driving enrichment of the TCR signalling gene set, including log_2_ fold change, nominal p-values, and FDR-adjusted q-values, highlighting the core contributors to pathway activation in aGvHD. (**D**) Pathway enrichment heatmaps derived from three independent annotation resources - WikiPathways, Reactome, and Gene Ontology. Each heatmap displays significantly enriched pathways differentiating aGvHD from non-aGvHD samples. Colour intensity reflects enrichment significance and directionality, with pathways enriched in aGvHD shown in red hues and those enriched in non-aGvHD samples shown in blue. (**E**) Cell-type enrichment analysis based on immune gene expression profiles. Dot plot illustrates the relative enrichment of predefined immune cell-type signatures across individual samples, highlighting cell populations associated with aGvHD pathology.

Inspection of differentially expressed immune-related genes (Figure 7A) highlighted transcriptional changes consistent with immune activation and altered tissue signalling in GI aGvHD. Among the most prominent features, IL13RA2 showed increased expression in aGvHD samples, whereas genes involved in antigen presentation, immune regulation, and extracellular matrix interactions, including FN1, CD74 and NOTCH1, were relatively reduced. These changes suggest a shift in immune–stromal interactions and antigen processing within inflamed GI tissue.

GSEA further demonstrated coordinated activation of immune signalling pathways rather than isolated gene-level effects. The most significantly enriched gene set was “Activation of the Pre-synaptic Phase of T-Cell Receptor (TCR) Signaling”, which showed a clear left-shifted enrichment score profile in aGvHD samples (Figure 7B). The leading edge subset driving this enrichment included genes such as IRF1, FCGR2A, CD74, CD163, and IL13RA2 (Figure 7C), all of which are implicated in antigen presentation, innate immune signalling, or inflammatory regulation, supporting enhanced immune activation in GI aGvHD tissue.

Pathway over-representation analysis across Reactome, WikiPathways, and Gene Ontology revealed convergent enrichment of pathways related to T cell activation, MHC class II-mediated antigen presentation, monocyte and macrophage migration, and immune response regulation (Figure 7D). The consistency of enriched pathways across multiple annotation platforms strengthens confidence in the biological relevance of these findings and highlights coordinated engagement of both innate and adaptive immune processes in GI aGvHD. Within these enriched pathways, IL13RA2 emerged as a recurrent upregulated contributor, while FN1, MR C1, and CD163 showed reduced expression, suggesting dysregulated macrophage-associated signalling and tissue remodelling during active disease.

Collectively, these analyses indicate that GI aGvHD is characterised by robust activation of immune signalling pathways, particularly those involving TCR signalling, antigen processing, and monocyte/macrophage-associated responses. These pathways nominate several candidate biomarkers and mechanistic drivers for further investigation, including IL13RA2, NOTCH1, and FN1.

### 2.7. Cell Type Enrichment Analysis Reveals Altered Myeloid Signatures in GI aGvHD

To explore the cellular context of transcriptional changes in GI aGvHD, we performed immune cell-type deconvolution using a computational approach based on immune gene expression signatures (Figure 7E). Contrary to initial interpretation, this analysis indicated depletion of M0 (undifferentiated) macrophage signatures in GI aGvHD samples, with relatively higher M0 macrophage-associated transcripts in GvHD-negative tissue. In contrast, both resting and activated dendritic cell signatures were relatively increased in GI aGvHD samples compared with controls.

Exploratory assessment suggested a trend towards higher dendritic cell signature scores in samples with higher histological aGvHD grade, although formal statistical correlations were limited by the small cohort size. While direct validation using spatial transcriptomics or immunohistochemistry was not performed, these findings align with pathway-level enrichment of monocyte and dendritic cell–associated processes and provide hypothesis-generating evidence that shifts in myeloid composition contribute to GI aGvHD pathology.

Collectively, these results provide hypothesis-generating evidence to indicate coordinated remodelling of myeloid cell transcriptional programs in inflamed GI tissue during aGvHD.

## 3. Discussion

Our NanoString profiling of microRNAs and immune-related genes in GI biopsies from allogeneic transplant recipients offers critical new insights into the molecular landscape of GI aGvHD. Focussing on local immune responses in the target organ itself, our comprehensive transcriptomic profiling identified a dysregulated network of immune-related genes and microRNAs associated with histologically confirmed aGvHD, revealing patterns of transcriptional suppression and post-transcriptional modulation with implications for both pathophysiology and clinical application. These results extend prior serum-based miRNA biomarker studies by defining site-specific molecular circuitry operative in inflamed GI mucosa.

Our expression profiling of GI biopsies revealed dominant upregulation of interleukins (such as IL1B and IL17RA), antigen-presentation molecules (HLA-DRA, CD74), and immune-regulatory microRNAs (miR-155-3p, miR-223-3p). These findings are in agreement with the cytokine-driven inflammation observed in previous aGvHD models and clinical serum studies. MiR-155 has been shown to drive GvHD pathogenesis via donor T-cell activation in systemic and murine models of aGvHD, including enhanced T cell expansion and inflammatory cytokine production [15,16,17]. Although these studies were not restricted to GI-restricted, the central role of donor T cell activation and cytokine amplification is shared across GvHD target organs, supporting relevance to intestinal disease. Similarly, miR-223 regulates innate immune activation and macrophage-driven inflammation across multiple inflammatory contexts [18,19], and its upregulation in GI aGvHD tissue may reflect conserved myeloid regulatory responses operating within the intestinal mucosa. Although direct mechanistic validation of these interactions was not performed in this study. Our conclusions are supported by convergent evidence from differential expression, machine learning prioritization, and pathway enrichment analyses, consistent with previously reported roles of these miRNAs in immune regulation. These data provide a strong rationale for future functional studies to confirm miRNA-target interactions and elucidate their contribution to GI aGvHD pathogenesis.

Our transcriptomic profiling of GI tissue in histologically confirmed GI aGvHD revealed distinct gene and microRNA expression changes that provide insight into the disease pathogenesis on a molecular level. Notably, several immune-regulatory and epithelial-associated genes and microRNAs were significantly dysregulated, highlighting disrupted immune homeostasis and barrier function in active GI aGvHD. Among the most significantly downregulated genes, ST6GAL1 and THBS1 emerged as strong candidates associated with GI aGvHD. ST6GAL1, a sialyltransferase involved in the glycosylation of epithelial and immune cell surface proteins, has been shown to preserve intestinal barrier integrity and suppress Th1/Th17 inflammation [20,21]. Its reduced expression is associated with increased mucosal inflammation in IBD and murine models [22,23]. Although these studies were conducted in inflammatory bowel disease and non-transplant models, they highlight epithelial and immune regulatory mechanisms that are directly relevant to GI aGvHD, where barrier disruption and Th1/Th17-skewed inflammation are central pathogenic features. Similarly, THBS1 (Thrombospondin-1) plays a multifaceted role in tissue repair, angiogenesis, and immune modulation via TGF-β activation, a key cytokine in gut immune homeostasis [24,25]. Reduced THBS1 expression may impair TGF-β signalling, destabilize epithelial junctions, and amplify dendritic cell activation, all of which are implicated in GI aGvHD pathogenesis [26,27,28]. While THBS1 has been predominantly studied in cancer and non-intestinal inflammatory contexts, its role in TGF-β activation, epithelial integrity, and dendritic cell regulation provides a mechanistic framework relevant to GI aGvHD, where impaired tolerogenic signalling contributes to mucosal injury. We also observed downregulation of several microRNAs implicated in epithelial integrity and immune regulation. Although many of these microRNAs have been characterised in non-GvHD inflammatory or epithelial injury models, their known molecular targets align with pathways central to GI aGvHD pathogenesis. MiR-1915-3p, which regulates apoptosis via Bcl-2 suppression, may contribute to increased epithelial cell death in GI aGvHD [29,30,31,32,33], likely contributes to epithelial dysfunction. MiR-4488, a putative suppressor of macrophage-mediated inflammation, may also support anti-inflammatory M2 phenotypes; its reduced expression may promote M1 polarization and cytokine release [34]. Further, downregulation of miR-197-3p, known to suppress IL1R1 and inflammasome activation, may exacerbate IL-1β–mediated inflammation in aGvHD [35,36]. Co-repressed microRNAs miR-143-3p and miR-145-5p, essential for intestinal homeostasis and cytokine modulation via NF-κB and TLR4 signalling [37,38,39,40] suggest loss of epithelial repair and tight junction regulation. Similarly, miR-146b-5p, which acts as a brake on NF-κB by targeting TRAF6 and IRAK1 [41,42], may be downregulated in GI aGvHD as part of unchecked innate immune activation. Lastly, miR-23b-3p, a known suppressor of NF-κB and STAT3 via direct targeting of TAB2/3 and IKK-α [43,44], was significantly decreased in GI aGvHD biopsies, potentially enabling heightened inflammatory gene expression and cytokine production.

Conversely, we identified increased expression of several pro-inflammatory genes and microRNAs in GI aGvHD. IRF1, a transcription factor downstream of interferon signalling, was significantly upregulated. Although IRF1 has been studied predominantly in systemic immune responses, its role in interferon-driven epithelial–immune crosstalk supports relevance to intestinal GvHD, where IFN-γ signalling is a key driver of mucosal damage. IRF1 regulates genes involved in antigen presentation, cytokine secretion, and T cell activation [45] and has been shown to promote Th1 differentiation and GvHD severity [46]. While IRF1 may also modulate GvL effects through IFNGR regulation [47], its increased expression in GI tissue likely reflects epithelial–immune cross-talk and IFN-γ–driven inflammation [48,49]. Among microRNAs, miR-223-3p was notably upregulated. A regulator of myeloid activation and NLRP3 inflammasome signalling [19,50], miR-223-3p has been shown to mitigate GvHD severity in murine models by suppressing pro-inflammatory cytokines and donor T cell infiltration [51]. Its elevated expression may therefore reflect a compensatory regulatory response to inflammation and may serve as a biomarker of local immune activation in the gut. We also observed upregulation of miR-137, which targets pro-inflammatory mediators such as SRC and CCL2 [52]. This may indicate a feedback mechanism attempting to limit tissue injury. Nonetheless, the context-dependent expression of such microRNAs highlights the complexity of immune regulation in GI aGvHD and underscores the potential for dynamic biomarker development.

Pathway analysis confirmed significant enrichment in IL-2/STAT5, IL-6/JAK/STAT3, TCR-signalling, complement activation, and antigen processing pathways. These signatures align with prior transcriptomic analyses of aGvHD in peripheral blood and gut tissue in murine and human studies [53]. Moreover, the upregulation of IL13RA2, a decoy receptor modulating IL-13 signalling, suggests potential dysregulation of epithelial repair and macrophage polarization processes, which have been linked to epithelial injury and macrophage polarisation in chronic GvHD and other inflammatory contexts [54], suggesting potential relevance to intestinal damage pathways operative in acute GI aGvHD. Our combined gene and microRNA network indicates an integrated regulatory circuit driving immune activation and tissue breakdown.

Machine learning–based ranking identified LCN2, CXCL13, MICB, CCL21, HLA-DQA1 and miR-1269b as top candidate tissue biomarkers, providing histological insight beyond prior serum studies (e.g., miR-29a) [55]. CXCL13, a chemokine involved in lymphocyte homing, has previously been reported as elevated in GvHD target tissues and blood [56,57]. LCN2 emerged as the most highly ranked candidate biomarker in histological GI aGvHD, with lower expression in aGvHD biopsies compared with no-GI-GvHD tissues. Prior studies indicate that LCN2-expressing intestinal neutrophils reduce aGvHD severity via IL-10 induction and suppression of MHCII in macrophages [58], suggesting a protective, tissue-specific role. This observed downregulation in active GI aGvHD may reflect loss or exhaustion of this regulatory axis, potentially due to neutrophil dysfunction or epithelial injury. Interestingly, plasma LCN2 has been reported to increase in aGvHD [59], highlighting a divergence between systemic and local tissue responses. Local downregulation of LCN2 may indicate dysregulated or depleted neutrophil function in severe disease, and its loss could contribute to uncontrolled inflammation in the gut. Together, these data support a model in which LCN2 plays a protective, tissue-specific role in early GvHD, with potential implications for prognosis and therapeutic intervention.

Gene set enrichment analysis confirmed that immune-related pathways were significantly overrepresented in GvHD samples. The top enriched gene set, derived from a GEO dataset of transporter genes expressed post-transplant (GSE102288), highlights parallels between our data and prior studies of transplant-related immune activation. The enrichment of this gene set suggests a conserved transcriptional signature in the post-transplant immune response, particularly involving genes linked to cellular trafficking, antigen processing, and tissue inflammation. Pathway enrichment analysis across WikiPathways, Reactome, and Gene Ontology databases further supported immune activation as a key hallmark of GI GvHD. Notably, there was significant enrichment of MHC class II antigen presentation, TNF-α and interferon signalling, and regulation of leukocyte-mediated cytotoxicity, all of which are critical pathways in alloreactivity and tissue. These pathways underscore the role of both innate and adaptive immune mechanisms, particularly macrophage and T cell effector functions, in the pathogenesis of intestinal GvHD.

Our deconvolution analyses identified reduced M0 macrophage-associated signatures and enrichment of resting and activated dendritic cell transcripts in GI aGvHD samples. This suggests active GI aGvHD is accompanied by remodelling of the myeloid compartment, with loss of undifferentiated macrophages and accumulation of dendritic cells. Exploratory trends indicated higher dendritic cell scores in samples with greater histological severity, although cohort size precluded formal statistical assessment. The depletion of M0 macrophages may reflect differentiation into pro-inflammatory subsets, consistent with prior functional studies of GvHD [60,61]. While direct spatial validation was not performed, these results provide hypothesis-generating evidence that shifts in myeloid transcriptional programs contribute to GI aGvHD pathology, complementing observed changes in miRNA expression and cytokine pathways. Further studies with larger cohorts and spatially resolved approaches are warranted.

Cell-type deconvolution also revealed a modest increase in CD8 T-cell signatures in a subset of two samples, providing a plausible cellular source for the elevated expression of cytotoxic effector molecules such as *GZMA* and *GZMB* observed in these biopsies. CD8 T cells are a well-established source of granzymes and play a central role in mediating epithelial injury in GI aGvHD [62]. Notably, both samples were derived from patients with underlying multiple myeloma, a condition associated with chronic immune dysregulation and altered T-cell composition, which may influence baseline immune contexture and contribute to inter-patient heterogeneity in cytotoxic signalling [63]. While the limited cohort size precludes formal stratification by underlying malignancy, these observations highlight the importance of considering pre-transplant diagnosis as a potential modifier of local immune responses in GI aGvHD and warrant further investigation in larger, disease-stratified cohorts.

Taken together, our integrated mRNA and microRNA profiling of GI biopsies from allogeneic transplant recipients with and without histologically confirmed GI aGvHD provides a comprehensive view of the immune and epithelial perturbations driving this complication. By examining gene and microRNA expression in matched biopsies at the site of tissue injury, we identified a regulatory network marked by cytokine-driven inflammation, disrupted antigen presentation, epithelial stress, and altered microRNA-mediated immune modulation. These changes implicate both innate and adaptive pathways, including macrophage and T cell activation, in the amplification of mucosal damage. Our approach offers a tissue-specific perspective not captured by previous serum- or blood-based biomarker studies. The concurrent upregulation of inflammatory genes (e.g., *IRF1*, *IL1B*), suppression of epithelial regulators (*ST6GAL1*, *THBS1*), and repression of homeostatic microRNAs (*miR-1915-3p*, *miR-145-5p*) suggest a breakdown in immune-epithelial crosstalk. Gene set enrichment and pathway analyses corroborated this, revealing activation of IL-1β, IFN-γ, and NF-κB signalling alongside impaired tolerogenic mechanisms. These findings support the concept of GI aGvHD as a localized immune-epithelial interface breakdown.

While this study provides novel insights into the transcriptomic changes in GI aGvHD, several limitations should be acknowledged. Firstly, the sample size, although clinically well-characterized, was relatively small and may restrict statistical power. Although pathway analysis and machine learning were used to mitigate overfitting, these analyses should be interpreted as exploratory and hypothesis-generating rather than definitive, and validation in larger, multicentre cohorts is essential. Future work should include functional studies to confirm key microRNA–mRNA interactions, as well as assessments of prognostic value for treatment response, particularly to steroids or targeted agents like ruxolitinib or vedolizumab. Secondly, as the analysis was performed on bulk tissue with only one biopsy per patient, it reflects an aggregate of diverse cellular populations within the gut mucosa, potentially masking cell-type-specific changes and increasing susceptibility to inter-individual variability. Future single-cell or spatial transcriptomic approaches could help disentangle these complex cellular dynamics. Furthermore, Temporal heterogeneity at biopsy collection may also influence expression patterns. Additionally, NanoString technology captures a targeted panel of transcripts, rather than broader transcriptomic changes detectable by RNA-seq. In addition, while RNAlater preservation facilitated RNA integrity, recent studies have demonstrated that it may introduce systematic biases in transcriptomic profiles, particularly affecting genes associated with osmotic stress and other functional categories [64]. These preservation-associated effects could influence pathway enrichment and interpretation of specific gene expression patterns. Lastly, although integration of mRNA and microRNA data allowed us to identify potential regulatory interactions, functional validation, such as luciferase reporter assays or in vitro knockdown/overexpression studies, is required to confirm causal relationships.

In conclusion, this study is the first to apply integrated mRNA-microRNA expression profiling, immune deconvolution, and pathway analysis directly to GI tissue from patients with histologically confirmed GI aGvHD. By interrogating gene and microRNA expression at the site of mucosal injury, we delineate a tissue-specific immune-epithelial landscape characterized by cytokine-driven inflammation, impaired epithelial homeostasis, and dysregulated immune regulation. These findings provide mechanistic insight into local disease biology that is not captured by peripheral blood or serum-based approaches and underscore the value of tissue-resolved profiling in GI aGvHD.

While this study identifies reproducible transcriptomic signatures and candidate molecules, including LCN2, CXCL13, and miR-223-3p, associated with active GI aGvHD, their diagnostic or prognostic utility has not yet been established. Clinical thresholds, temporal dynamics, and monitoring strategies remain undefined. As such, these findings should be viewed as hypothesis-generating rather than immediately translatable. Future prospective studies incorporating larger, independent cohorts, spatial or single-cell transcriptomics, and correlation with clinical outcomes will be required to determine whether these tissue-derived biomarkers can inform risk stratification, treatment response, or disease monitoring in GI aGvHD.

## 4. Materials and Methods

### 4.1. Clinical Samples and Ethics

A total of n = 8 GI biopsy samples were included in the NanoString analyses, and comprised a single biopsy per patient from one colonic region. Of these, n = 3 samples were classified as GI aGvHD-positive (histological grade II-IV) and n = 5 as GI aGvHD-negative (histological grade 0-I). All patients underwent allogeneic HSCT at University Medical Centre, Regensburg, between 2010 and 2013 (Table 1). Clinical metadata, including overall survival, relapse status, clinical aGvHD stage, and histological grade, were available for all included samples (Table 2).

All patients provided informed consent for sample collection and molecular analyses. The project was approved by the University of Regensburg Ethics Commission (Application 17-618-101) and conducted in accordance with the Declaration of Helsinki. Clinical diagnosis and grading of aGvHD were performed by treating clinicians in accordance with the NIH consensus and modified Glucksberg criteria [5,65]. All clinical data were obtained from the EuroTransplantBank database (www.EuroTransplantBank.org).

### 4.2. Total RNA Isolation

RNA was extracted from the GI biopsy samples stored in RNAlater (Ambion, Waltham, MA, USA) using the *mi*rVana microRNA Isolation Kit (Ambion) according to the manufacturer’s recommendations and quantified using the NanoDrop ND-1000 spectrophotometer (Thermo Scientific, Waltham, MA, USA). cDNA was generated by random hexamer priming. Briefly, equal quantities of RNA and 2 x strength cDNA generation mix containing random hexamer primers (Pharmacia, Marlborough, MA, USA), dNTPs (Roche, Basel, Switzerland), reverse transcriptase (Life Technologies, Paisley, UK) and an RNase inhibitor (RNasin, Promega, Madison, WI, USA) were incubated together at 37 °C for 2 h, with a further incubation at 65 °C for 10 min to denature the reverse transcriptase.

### 4.3. NanoString mRNA and MicroRNA Profiling

Total RNA was profiled using the nCounter^®^ Human PanCancer Immune Profiling Panel (NanoString Technologies, Seattle, WA, USA) and the nCounter^®^ Human v3.0 microRNA Expression Assay (NanoString Technologies), based on miRBase v21. For both assays, 150 ng of total RNA was used as starting material.

The PanCancer Immune Profiling Panel comprises 770 immune-related genes, including 109 cell surface markers representing 24 immune cell types, 30 cancer–testis antigens, and over 500 genes involved in immune response pathways. The microRNA assay profiles 799 mature human microRNAs and includes built-in positive, negative, ligation, and mRNA housekeeping controls (ACTB, B2M, GAPDH, RPL19, and RPLP0).

### 4.4. NanoString Data Preprocessing, QC and Analysis

Raw NanoString count data were subjected to standard preprocessing using nSolver Analysis Software v2.5 (NanoString Technologies). Initial quality control was performed according to manufacturer guidelines, including assessment of imaging quality, binding density, positive control linearity, and limit-of-detection (LOD) thresholds. All samples included in downstream analyses met NanoString-recommended QC criteria.

Background correction was performed using the mean plus two standard deviations of the negative control probes within each sample. Probes with expression values below the calculated LOD in the majority of samples were retained for exploratory analyses but were excluded from differential expression and enrichment analyses to reduce background-driven noise.

For the mRNA dataset, codeset content normalization was carried out using the geometric mean of 40 endogenous housekeeping genes. For the microRNA dataset, normalization was performed using the geometric mean of the top 100 most highly expressed microRNAs, as implemented in nSolver. Normalized data were log2-transformed prior to downstream statistical analyses.

Following normalization, the total number of samples was annotated according to clinical and pathological parameters. Samples were categorised by gut aGvHD status (No: histological grade 0–I; Yes: histological grade II–IV), overall survival (Alive or Dead), relapse status (Yes or No), clinical aGvHD stage (grades 1–3), and histological aGvHD grade (grades 0–4) [66].

### 4.5. Statistical Analysis

Given the limited cohort size, a conservative analytical strategy was adopted to minimise false discovery. Where applicable, only features identified as significant across multiple independent statistical approaches were retained. To account for latent technical or biological sources of variation, unsupervised batch correction was performed on normalized expression data using surrogate variable analysis (SVA). Estimated surrogate variables were regressed out using the ‘removeBatchEffect’ function from the limma R (v3.62.2)/Bioconductor (v3.20) package. Associations between phenotypes were evaluated according to data type. Fisher’s exact test was used for categorical–categorical comparisons, Kruskal–Wallis tests for categorical–continuous comparisons, and Pearson correlation for continuous–continuous comparisons. Unsupervised clustering and heatmap visualisation were performed using the ComplexHeatmap R/Bioconductor package (v3.20) on scaled log-expression values (z-scores), using Euclidean distance and Ward’s linkage [67]. Genes and microRNAs were selected for clustering based on standard deviation. In heatmaps, red indicates relative overexpression and blue indicates relative underexpression. Functional annotation of expression clusters was performed by ranking enrichment of gene sets from MSigDB [68], Gene Ontology [69], and KEGG [70]. using correlation-based approaches. Clustering was implemented using the fastcluster R package (v 1.01-2) [71]. Dimensionality reduction was performed using Uniform Manifold Approximation and Projection (UMAP). The top 1000 most variable genes were first reduced to 50 principal components, followed by UMAP embedding using the uwot R package (version 0.2). The number of neighbours was set heuristically to 25% of the sample size (minimum 2, maximum 30). Differential expression analysis was performed using three independent statistical frameworks: DESeq2 (Wald test) [72], edgeR (quasi-likelihood F test) [73], and limma-trend [74]. For each feature, the maximum adjusted q-value across the three methods was retained as the aggregate q-value, thereby defining significance based on concordance across methods. To assess variable importance and identify candidate biomarkers, multiple machine learning approaches were applied, including sparse partial least squares (sPLS), elastic net regression [75], random forest [76], and extreme gradient boosting [77]. Features were ranked based on cumulative importance scores across models. Gene set enrichment analyses were conducted using CAMERA [78], GSEA [79], ssGSEA [80], fGSEA [81], GSVA [82], and fry [74]. Enrichment q-values from individual methods were combined by retaining the maximum value as a meta-q value. Cell type deconvolution was performed using the LM22 signature matrix as a reference dataset [83].

## Figures and Tables

**Table 1:** Clinical Details for NanoString Profiling GI aGvHD Samples. RNA from eight post-HSCT GI tissue biopsies (three aGvHD, five no GvHD) was profiled. Pt = Patient; D = Donor; Cond. = Conditioning; Rel. = Relationship; F = Female; M = Male; CMV = cytomegalovirus; Tx = Transplant; OS = Overall survival; RIC = Reduced intensity conditioning; SIB = Sibling; MUD = Matched unrelated donor; AML = Acute myeloid leukemia; MM = Multiple myeloma; NHL = Non-Hodgkin’s lymphoma; CLL = Chronic lymphocytic leukemia; MDS = Myelodysplastic syndrome; CR = Complete remission; PR = Partial remission; Prog = Progression; A = Alive; D = Deceased.

ID	Pt Gend.	D Gend.	Cond.	Rel.	P Age	D Age	CMV	Disease	State	Tx	OS	Relapse	Histo aGvHD	NanoString
1	M	F	RIC	SIB	56	59	1	AML	CR1	2010	A	N	II	mRNA & microRNA
2	M	M	RIC	MUD	56	34	1	MM	PR1	2010	D	Y	0	mRNA & microRNA
3	F	M	RIC	MUD	24	41	0	AML	Rel	2012	D	N	IV	mRNA & microRNA
4	F	M	RIC	MUD	63	35	0	AML	Other	2010	D	N	II	mRNA & microRNA
5	M	M	RIC	MUD	61	43	1	NHL	Prog	2013	A	N	I	mRNA & microRNA
6	M	M	RIC	MUD	44	40	1	MM	PR1	2011	A	Y	0	mRNA & microRNA
7	M	M	RIC	MUD	51	35	1	MDS	Other	2011	A	N	0	mRNA & microRNA
8	M	M	RIC	MUD	45	34	0	CLL	PR1	2010	D	Y	I	mRNA & microRNA

**Table 2:** Clinical and Demographic Details of the Clinical Cohort. Clinical details of the patient cohort (n = 8). *p*-values between groups were calculated using the ^1^ independent 2-sample *t*-test or ^2^ Fisher’s Exact test, as appropriate. Patients received HSCT for a range of underlying conditions, including multiple myeloma (n = 2), acute myeloid leukemia (n = 7), chronic lymphocytic leukemia (n = 1), Non-Hodgkin’s lymphoma (n = 4), and myelodysplastic syndrome (n = 1). MUD = matched unrelated donor; SIB = sibling donor; RIC = Reduced. intensity conditioning; CMV = Cytomegalovirus.

	All	No GvHD (N, %)	aGvHD (N, %)	*p*-Value
**All**	8	5	3	
**Patient gender ^2^**				
Male	6	5 (100)	1 (34)	0.11 **^2^**
Female	2	0 (0)	2 (66)	
**Donor gender ^2^**				
Male	7	5 (100)	2 (66)	0.38 **^2^**
Female	1	0 (0)	1 (34)	
**CMV Status ^2^**				
Positive	5	4 (80)	1 (34)	0.46 **^2^**
Negative	3	1 (20)	2 (66)	
**Av. Age ^1^**	50	48	51	1.00 **^1^**
Range	24–63	44–61	24–63	
**Relationship ^2^**				
MUD	7	5 (100)	2 (66)	0.34 **^2^**
SIB	1	0 (0)	1 (34)	
**Outcome ^2^**				
Alive	4	3 (60)	1 (34)	1.00 **^2^**
Dead	4	2 (40)	2 (66)	

## Data Availability

The raw data supporting the conclusions of this article will be made available by the authors on request.
